# Genetic associations of in vivo pathology influence Alzheimer’s disease susceptibility

**DOI:** 10.1186/s13195-020-00722-2

**Published:** 2020-11-19

**Authors:** Jieun Seo, Min Soo Byun, Dahyun Yi, Jun Ho Lee, So Yeon Jeon, Seong A. Shin, Yu Kyeong Kim, Koung Mi Kang, Chul-Ho Sohn, Gijung Jung, Jong-Chan Park, Sun-Ho Han, Jayoung Byun, Inhee Mook-Jung, Dong Young Lee, Murim Choi, for the KBASE Research Group

**Affiliations:** 1 Department of Biomedical Sciences, Seoul National University College of Medicine, Seoul, Republic of Korea; 2 Department of Neuropsychiatry, Seoul National University Bundang Hospital, Gyeonggi, Republic of Korea; 3 Institute of Human Behavioral Medicine, Medical Research Center Seoul National University, Seoul, Republic of Korea; 4 Department of Neuropsychiatry, National Center for Mental Health, Seoul, Republic of Korea; 5 Department of Psychiatry, Chungnam National University Hospital, Daejeon, Republic of Korea; 6 Department of Nuclear Medicine, SMG-SNU Boramae Medical Center, Seoul, Republic of Korea; 7 Department of Radiology, Seoul National University Hospital, Seoul, Republic of Korea; 8 Department of Neuropsychiatry, Seoul National University Hospital, Seoul, Republic of Korea; 9 Department of Biochemistry, Seoul National University College of Medicine, Seoul, Republic of Korea; 10 Department of Medicine, Pusan National University, Busan, Republic of Korea; 11 Department of Psychiatry, Seoul National University College of Medicine, Seoul, Republic of Korea

**Keywords:** Alzheimer’s disease, Targeted panel sequencing, Genetic association, Neuroimaging, In vivo AD pathologies, PET, MRI

## Abstract

**Introduction:**

Although the heritability of sporadic Alzheimer’s disease (AD) is estimated to be 60–80%, addressing the genetic contribution to AD risk still remains elusive. More specifically, it remains unclear whether genetic variants are able to affect neurodegenerative brain features that can be addressed by in vivo imaging techniques.

**Methods:**

Targeted sequencing analysis of the coding and UTR regions of 132 AD susceptibility genes was performed. Neuroimaging data using ^11^C-Pittsburgh Compound B positron emission tomography (PET), ^18^F-fluorodeoxyglucose PET, and MRI that are available from the KBASE (Korean Brain Aging Study for Early Diagnosis and Prediction of Alzheimer’s disease) cohort were acquired. A total of 557 participants consisted of 336 cognitively normal (CN) adults, 137 mild cognitive impairment (MCI), and 84 AD dementia (ADD) groups.

**Results:**

We called 5391 high-quality single nucleotide variants (SNVs) on AD susceptibility genes and selected significant associations between variants and five in vivo AD pathologies: (1) amyloid β (Aβ) deposition, (2) AD-signature region cerebral glucose metabolism (AD-Cm), (3) posterior cingulate cortex (PCC) cerebral glucose metabolism (PCC-Cm), (4) AD-signature region cortical thickness (AD-Ct), and (5) hippocampal volume (Hv). The association analysis for common variants (allele frequency (AF) > 0.05) yielded several novel loci associated with Aβ deposition (*PIWIL1-*rs10848087), AD-Cm (*NME8*-rs2722372 and *PSEN2*-rs75733498), AD-Ct (*PSEN1-*rs7523) and, Hv (*CASS4-*rs3746625). Meanwhile, in a gene-based analysis for rare variants (AF < 0.05), cases carrying rare variants in *LPL*, *FERMT2*, *NFAT5*, *DSG2*, and *ITPR1* displayed associations with the neuroimaging features. Exploratory voxel-based brain morphometry between the variant carriers and non-carriers was performed subsequently. Finally, we document a strong association of previously reported *APOE* variants with the in vivo AD pathologies and demonstrate that the variants exert a causal effect on AD susceptibility via neuroimaging features.

**Conclusions:**

This study provides novel associations of genetic factors to Aβ accumulation and AD-related neurodegeneration to influence AD susceptibility.

**Supplementary information:**

**Supplementary information** accompanies this paper at 10.1186/s13195-020-00722-2.

## Background

Alzheimer’s disease (AD), the most common cause of dementia, is a neurodegenerative disease with high heritability estimated to be 60–80% [1, 2]. Previous genetic studies have elucidated causative rare variants contributing to familial AD that commonly occurs before the age of 65 (early onset). These variants are found in genes related to amyloid beta (Aβ) synthesis, such as *APP* (amyloid precursor protein), *PSEN1* (presenilin 1), and *PSEN2* (presenilin 2) [3]. However, as early-onset familial AD accounts for < 5% of AD cases [2, 3], unraveling the complex genetic contributions to sporadic AD cases, which represents the majority of AD cases that occur after age 65 (late onset), is important. To date, apolipoprotein E (*APOE*) ε4 allele (*APOE4*) is the strongest genetic factor of sporadic AD [4], which confers a 3- to 15-fold increased risk of AD [5]. Recent large-scale genome-wide association studies (GWAS) have identified novel common variants of AD in loci involved in amyloid synthesis, nervous system development, synaptic transmission, and inflammation pathways [4, 6–9]. Nevertheless, *APOE4* and several other common variants identified by previous GWAS explain a modest fraction of AD heritability [4], and addressing the remainder still remains as a challenge.

Most of the previous GWAS relied on phenotypes defined by clinical diagnosis of AD dementia (ADD) [8] and not by AD pathology or its surrogate biomarkers*.* Therefore, it remains unclear whether previously reported genes exert associations through modulating in vivo AD pathologies. Also, applying next-generation sequencing (NGS) technologies increases the possibility of detecting rare variants with large effect on diseases, which may help to explain the missing heritability [2, 10]. Although recent efforts to aggressively integrate genome-wide effects of common and rare variants has substantially improved our understanding of AD genetics [11, 12], how individual variants function to confer such effects still requires further investigation. A number of previous studies investigated genetic association with in vivo AD pathologies using AD biomarkers, but none has utilized multiple in vivo AD pathology features and integrated them with common and rare genetic variants [13–18].

Although genome-wide approaches enable unbiased screening of novel variants, we chose to perform a targeted sequencing (TS) approach as it provided the following advantages. First, overarching hypothesis of the study assumes that there are quantifiable contributions of imaging biomarkers on the known genetic signals for LOAD, thereby aiming to provide clues of variant functions. Therefore, we decided to focus our analyses on the selected 132 genes with previous associations with LOAD. Second, compared to genome-wide approaches, the number of data points in TS is substantially smaller and allows reduced statistical penalty. Lastly, TS reduces sequencing cost and allows easier data handling.

Based on an imaging genetics approach, this work aimed to identify common and rare genetic variants closely associated with cerebral Aβ deposition and AD-type neurodegeneration by conducting a TS analysis of 132 AD-related genes in 557 deep-phenotyped participants with multi-modal brain imaging information including [^11^C] Pittsburgh Compound B (PiB)-positron emission tomography (PET), [^18^F] fluorodeoxyglucose (FDG)-PET, and magnetic resonance imaging (MRI). Our multivariable analysis approach provides a more accurate model for AD susceptibility by comparing with biomarkers for in vivo AD pathology.

## Materials and methods

### Participants

We evaluated 557 participants who were recruited by the Korean Brain Aging Study for the Early Diagnosis and Prediction of Alzheimer’s disease (KBASE), a prospective cohort study initiated in 2014 (Supplementary Table S1) [13]. Participants consisted of 274 cognitively normal (CN) older adults (CN-old, age ≥ 55), 62 CN young and middle-aged adult (CN-ym, age < 55), 137 individuals with mild cognitive impairment (MCI), and 84 patients with ADD. Details on the recruitment and inclusion/exclusion criteria are described in the Supplementary Methods.

### Evaluation of in vivo AD pathologies

All participants underwent comprehensive clinical and neuropsychological evaluations and multi-modal brain imaging (including [^11^C]PiB-PET, [^18^F]FDG-PET, and MRI). Blood was sampled for DNA extraction. *APOE* genotyping was conducted as previously described [18]. More detailed information on the standardized assessment used in the KBASE cohort is described in a previous report [13]. For surrogate markers of in vivo cerebral Aβ deposition, we quantified the standardized uptake value ratio (SUVR) of global [^11^C] PiB retention level in the cortical region-of-interests (ROIs). [^18^F]FDG-PET was used to measure cerebral glucose metabolism in the AD-signature regions (AD-Cm) and posterior cingulate cortex (PCC-cm), which can capture early regional metabolic and functional deficits related to AD processes [14, 15]. T1 MRI was used to evaluate cortical thickness in the AD-signature regions (AD-Ct) [19] and intracranial volume-adjusted hippocampal volume (Hv) [16] based on previous studies. Details of acquisition and preprocessing of [^11^C]PiB-PET, [^18^F]FDG-PET, and MRI and the definition of each AD imaging biomarker are provided in the Supplementary Methods and Supplementary Table S2.

### AD gene panel, sequencing, and variant calling

The 132 genes were selected according to one of the following criteria: (1) genes from previous AD GWAS (*n* = 32) [6–9], (2) genes with mutations reported to be causative for familial AD in the OMIM database (*n* = 19), (3) genes with biological relevance in AD in the KEGG pathway (hsa05010) (*n* = 53), or (4) manually selected genes with potential relevance in AD (*n* = 28) (Supplementary Table S3). The AD gene panel was designed using the Ion AmpliSeq Designer (http://ampliseq.com) and contained 5049 amplicons with sizes ranging from 125 to 275 bp. The target regions included UTR and coding exons with 10 bases of padding sequences, totaling 1.46 Mb and covering 96% of the targeted intervals. AD-targeted panel sequencing was performed with an Ion Proton sequencer (Supplementary Methods, Supplementary Fig. S1 and Supplementary Table S4). The reads were aligned to hg19 and SNVs were called using Torrent Suite v3.4.2. Variants with < 20× coverage, located in homopolymer repeats, or covered < 90% of the samples were excluded. After applying the variant filtering criteria established in our pilot experiments (Supplementary Methods and Supplementary Fig. S1), 5391 variants remained.

### Association tests

Both continuous and categorical variables indicating in vivo AD pathologies were normalized and used as dependent variables to identify common variants (allele frequency (AF) > 0.05) associated with bioimaging markers (Supplementary Methods and Supplementary Table S2). Age, sex, and the number of *APOE4* alleles were used as covariates. Significant associations were defined at an uncorrected *P* < 0.001, following the previous studies [20–22], and the Benjamini and Hochberg method was used for multiple test correction for *APOE* variant association tests (Supplementary Table S5). Among the rare variants (AF < 0.05), loss-of-function (LoF) variants and missense variants with strong evolutionary conservation were selected for gene-based association analyses. R (version 3.5.1) was used to conduct Fisher’s exact test with the five binominal neuroimaging traits (Supplementary Table S2) and cognitive impairment, and rare variant enrichment across each gene was adjusted by length. For *APOE* variants, variants with AF > 0.1 were selected and tested for association, after adjusting for age and sex. For conditional analysis, a quantitative imaging trait was used as a covariate in an association test of *APOE* variants and AD susceptibility.

### Voxel-based analysis of multi-modal brain imaging

To identify regional PiB uptake differences, Statistical Parametric Mapping 12 (SPM12; http://www.fil.ion.ucl.ac.uk/spm) was used for the exploratory voxel-based analysis of [^11^C]PiB-PET. Similarly, voxel-based analyses of [^18^F]FDG-PET and T1 MRI were performed to visualize the differences of regional cerebral glucose metabolism or gray matter (GM) density by the presence of variants associated with AD-Cm, PCC-Cm, AD-Ct, or Hv. Additional details of the voxel-wise analysis of common variants and genes with rare variants are provided in the Supplementary Methods.

## Results

### Establishment of the AD panel, quality assessment, and variant calling strategy

**Fig. 1:**
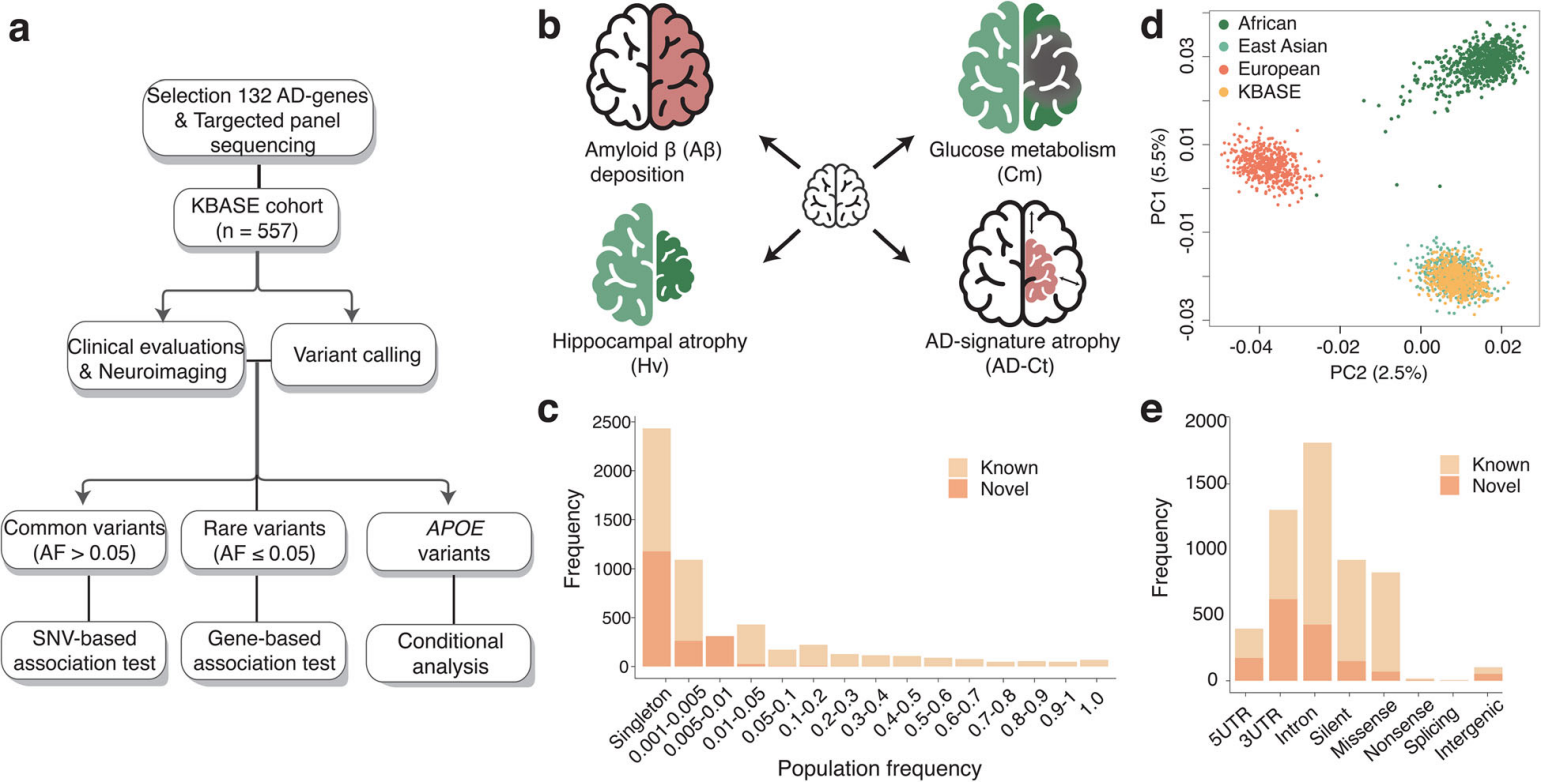
Study design and variant profile of the cohort. **a** Flowchart of the study design. **b** Phenotyping strategies of in vivo AD pathology. **c** Principal component analysis (PCA) of the KBASE cohort with individuals from the 1000 Genomes Project individuals across different populations. **d** Distribution of variants in the KBASE cohort by population frequency. **e** Distribution of variants by genetic regions

The overall study design and documented in vivo pathology biomarkers are represented in Fig. 1a, b. The clinical and demographic characteristics of the 557 participants are described in Supplementary Table S1. The mean age of the participants is 67.4 years, and 57.8% are women. The frequency of the *APOE4* allele in each diagnostic group indicated a strong disease association (i.e., 9.3% and 35.7% for CN-old and dementia groups, respectively). The mean coverage depth of the AD target sequencing runs was 436.8×, and 97.8% of the targeted bases were covered more than 20×, ensuring high variant sensitivity (Supplementary Table S4).

We developed an in-house SNV calling method for ion proton sequencing data. Prior to data production, we optimized the variant calling pipeline through a series of pilot experiments and demonstrated 92.4% sensitivity and 93.5% specificity (Supplementary Fig. S1). To further validate the quality of the 5391 called variants, principal component analysis (PCA) of the KBASE cohort was performed with individuals from the 1000 Genomes Project, which showed that our cohort co-clustered with the East Asian group (Fig. 1c). A strong correlation of AFs between our cohort and 1000 Genomes East Asian was observed (Pearson’s *r* = 0.99 [95% CI, 0.989–0.991]). The majority of the variants (79.2%) are rare (AF < 0.05), and 45.2% were singletons (seen once in our cohort; Fig. 1d). The majority of the variants (88.9%) with an AF > 0.001 are already listed in the 1000 Genomes, The Genome Aggregation Database (gnomAD), and the Korean Variant Archive (KOVA) [23–25]. The variants are composed of 31.5% UTR, 1.9% intergenic, 33.7% intronic, and 32.9% coding region locations (Fig. 1e). Each individual carried an average of 369.0 heterozygous variants, 295.4 homozygous variants, and 4.4 singletons. The mean ratio of missense-to-silent variants was 0.44, and the mean ratio of transition-to-transversion variants was 2.83 per individual, comparable to other large-scale genome data sets [26].

### Common variant association test

Tests that evaluated the association between common variants and Aβ deposition, AD-Cm, PCC-Cm, AD-Ct, and Hv revealed seven loci with strong associations, of which six are novel (Table 1). The CN-ym group was excluded from the test, as this group was entirely negative for the in vivo AD imaging biomarkers regardless of genotype.

**Table 1:** List of common variants that significantly associated with brain imaging features (*P* < 1.0 × 10^−3^)

AD imaging biomarker	Data type	Chr:position (hg19)	dbSNP ID	Gene^a^	PF^b^	Previously reported	*P*
I. Cerebral amyloid-β accumulation measured by PiB-PET							
Aβ deposition	Bin.^c^	chr3:39138840	rs3732377	*GORASP1*	0.237	Novel	9.32 × 10^−4^
		chr3:39139776	rs1109643	*GORASP1*	0.161	Novel	9.79 × 10^−4^
		chr3:39149277	rs28362644	*GORASP1*	0.162	Novel	7.02 × 10^−4^
		chr11:47345916	rs2290149	*MADD*	0.082	Novel	2.02 × 10^−4^
	Quant.^d^	chr12:130839165	rs10848087	*PIWIL1*	0.105	Novel	5.05 × 10^−4^
II. Glucose metabolism levels measured by FDG-PET							
AD-Cm	Bin.	chr1:227077809	rs75733498	*PSEN2*	0.083	Novel	1.75 × 10^−4^
	Quant.	chr7:37890267	rs2722372	*NME8*	0.191	Novel	7.63 × 10^−4^
PCC-Cm	Quant.	chr7:37890267	rs2722372	*NME8*	0.191	Novel	5.71 × 10^−4^
III. Cortical thickness measured by MRI							
AD-Ct	Bin.	chr14:73686944	rs7523	*PSEN1*	0.161	Novel	1.74 × 10^−5^
	Quant.	chr12:130839165	rs10848087	*PIWIL1*	0.105	Novel	2.94 × 10^−4^
IV. Hippocampal volume reduction measured by MRI							
Hv	Quant.	chr20:55033476	rs3746623	*CASS4*	0.945	Novel	1.73 × 10^−4^
		chr20:55033647	rs3746625	*CASS4*	0.946	Novel	1.73 × 10^−4^
		chr20:55033713	rs3746626	*CASS4*	0.946	Novel	1.73 × 10^−4^
		chr20:55033856	rs4811697	*CASS4*	0.946	Known [27]	3.42 × 10^−4^
	Both	chr12:130839165	rs10848087	*PIWIL1*	0.105	Novel	4.24 × 10^−4^

^a^The most closely located gene from each variant

^b^Population frequency in the KBASE cohort

^c^Categorical variable trait transformed from normalized neuroimaging data by each criterion

^d^Quantitative normalized neuroimaging variable trait

**Fig. 2:**
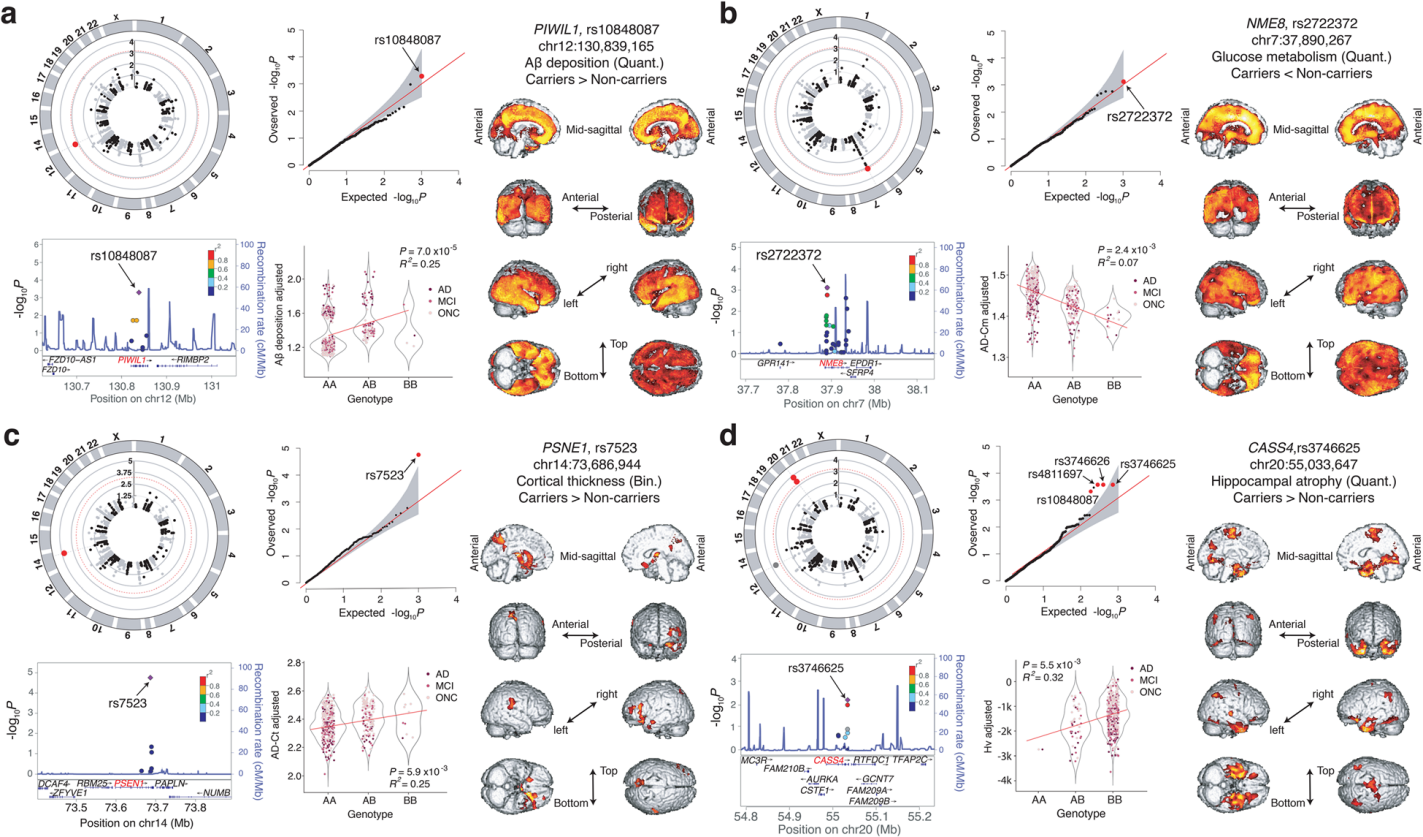
Common variants that are significantly associated with neuroimaging features. For each signal, a circular Manhattan plot, quantile-quantile (Q-Q) plot, regional plot, regression plot with adjusted trait values, and voxel-based clustering analysis result are shown. **a** rs10848087 in *PIWIL1* with cerebral Aβ deposition in global brain regions. **b** rs2722372 in *NME8* with AD-Cm. **c** rs7523 in *PSEN1* with AD-Ct. **d** rs4811697 in *CASS4* with Hv

Variants in *GORASP1* (rs28362644; odds ratio [OR] 0.46 [0.29–0.72]; *P* = 7.0 × 10^−4^), *MADD* (rs2290149; OR 2.74 [1.61–4.68]; *P* = 2.0 × 10^−4^), and *PIWIL1* (rs10848087; regression coefficient [*β*] 0.14 [0.06–0.22]; *P* = 5.1 × 10^−4^) were significantly associated with Aβ deposition (Table 1; Fig. 2a; Supplementary Fig. S2a). Comparison with voxel-based imaging data (*P* < 0.01, *k* = 1497) revealed that carriers of *MADD-*rs2290149 displayed greater Aβ deposition mainly in the bilateral, lateral, and medial frontal cortices; cingulate; precuneus; and lateral temporal and inferior parietal regions. *PIWIL1*-rs10848087 non-carriers showed diffuse Aβ accumulation in the bilateral cerebral cortices including the frontal, temporal, parietal, occipital lobes and basal ganglia (Fig. 2a). Three SNVs near *GORASP1* were identified, located in the same linkage disequilibrium (LD) block (*R*^2^ = 0.98; *D′* = 0.99), and the most robust SNP, rs28362644, was used for imaging analysis (Table 1). *GORASP1*-rs28362644 carriers showed less Aβ deposition in the posterior hippocampal and parahippocampal regions, as well as retrosplenial and precuneus regions at a more lenient threshold compared to non-carriers (*P* < 0.05, *k* = 5058; Supplementary Fig. S2a).

Two novel significant associations with AD-Cm were observed: *PSEN2-*rs75733498 (OR 0.39 [0.23–0.63]; *P* = 1.8 × 10^−4^) and *NME8-*rs2722372 (*β* − 0.04 [− 0.06 to − 0.018]; *P* = 7.6 × 10^−4^) (Table 1; Fig. 2b; Supplementary Fig. S2b). Subsequent voxel-based FDG-PET analysis revealed that *PSEN2-*rs75733498 non-carriers had reduced glucose metabolism levels in the bilateral fronto-temporo-parietal cortices (*P* < 0.01, *k* = 1497; Fig. 2b). On the other hand, *NME8*-rs2722372 carriers displayed diffuse cerebral glucose hypometabolism in the bilateral fronto-temporo-parietal cortices and subcortical structures, such as the thalamus and basal ganglia (Fig. 2b). An association between AD-Cm and *NME8*-rs2722372 in the PCC was also observed.

*PSEN1-*rs7523 (OR 0.39 [0.25–0.60]; *P* = 1.7 × 10^−5^) and *PIWIL1-*rs10848087 (*β* − 0.07 [− 0.11 to − 0.03]; *P* = 2.9 × 10^−4^) were associated with AD-Ct (Table 1; Fig. 2c; Supplementary Fig. S2c). Exploratory voxel-based analysis of T1 MRI demonstrated that *PSEN1-*rs7523 non-carriers showed reduced cortical thicknesses as compared to carriers in the inferior frontal, orbitofrontal, and basal ganglia (*P* = 2.5 × 10^−5^, *k* = 1497; Fig. 2d).

Finally, *CASS4-*rs3746625 (*β* 0.54 [0.26–0.82]; *P* = 1.7 × 10^−4^) and *PIWIL1-*rs10848087 (*β* − 0.40 [− 0.61 to − 0.19]; *P* = 2.3 × 10^−4^) strongly correlated with Hv (Table 1; Fig. 2d; Supplementary Fig. S2c). There are also three other significant SNVs in *CASS4* that are located in the same LD block with rs3746625 (*R*^2^ = 1.00; *D′* = 1.00). Brain images showed that GM atrophy was reduced in rs3746625 carriers than non-carriers, particularly in the bilateral medial temporal regions including the hippocampi (*P* < 0.01, *k* = 1497; Fig. 2d). To elucidate the functional implication of the *CASS4* and *PIWIL1* variants in the brain, ChIP-seq data from the ENCODE were utilized [28]. Notably, H3K27me3 signals, which mark heterochromatin, encompass the variants specifically in the hippocampus, but not in other parts of the brain or other organs, consistent with the results of voxel-based analysis of MRI (Supplementary Fig. S3).

The cumulative effect of common variants associated with the imaging biomarkers was evaluated by correlating AD risk score, defined as the weighted sum of the effect sizes of common variants associated with imaging features and the proportion of individuals with cognitive impairment (i.e., MCI or ADD; Supplementary Fig. S4). Common SNVs associated with each imaging biomarker (*P* < 0.05) explained 27–58% of cognitive impairment and 76% when used in combination.

### Effect of APOE variants

**Fig. 3:**
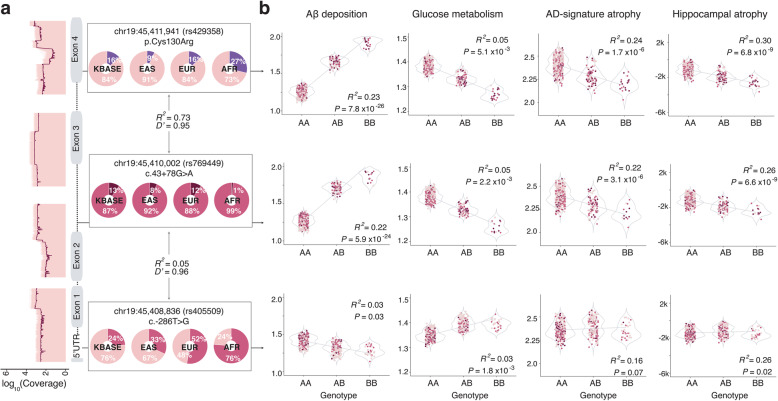
Association of *APOE* variants with AD imaging biomarkers. **a** Log10-scaled coverage map of *APOE* in the KBASE cohort, along with the gene structure shown with gray boxes indicating exons. Black lines indicate the average coverage depths. On the right, AF of the three *APOE* variants in KBASE and major ethnic groups are displayed. **b** Regression plots for the three variant genotypes and imaging traits after adjusted with age and sex

The *APOE4* allele possesses the strongest association with AD susceptibility [8], but comprehensive analysis of its impact on structural and functional changes in a large number of aging brains remains understudied. In addition, our study design allowed association assessment of other *APOE* variants. Therefore, we sought to identify association between *APOE* common variants and the imaging features. Rare variants were not detected. We tested three common variants with an AF > 0.1. Two variants, rs429358 and rs769449, are located in the same LD block (*R*^2^ = 0.73, *D′* = 0.95; Fig. 3a) and are strongly correlated with all five imaging traits (Table 2; Fig. 3b). An additional variant (rs405509) located in the promoter is in weak LD with rs429358 (*R*^2^ = 0.05, *D′* = 1.00) and rs769449 (*R*^2^ = 0.06, *D′* = 0.96; Fig. 3a). rs405509 possessed minimal association with the brain features (Table 2), with the association specifically residing in the 3′-portion of the gene.

**Table 2:** Effects of common *APOE* SNVs on imaging biomarkers and conditional analysis of the SNVs controlling for each imaging biomarkers

*APOE* SNP	Imaging biomarkers	Association (Quant.)			Cognitive function association	
		*β*	*P*	*P*_adj_	Unconditioned	Controlling for imaging biomarker (Quant.)
rs429358 (chr19:45411941)	Aβ deposition	0.37	2.85 × 10^−27^	2.89 × 10^−24^	2.03 × 10^−9^	0.047
	AD-Cm	− 0.06	5.61 × 10^−6^	2.85 × 10^−3^		2.18 × 10^−5^
	PCC-Cm	− 0.10	3.07 × 10^−10^	3.12 × 10^−7^		3.16 × 10^−3^
	AD-Ct	− 0.11	8.70 × 10^−13^	8.80 × 10^−10^		2.82 × 10^−3^
	Hv	− 0.78	2.23 × 10^−18^	2.25 × 10^−15^		0.150
rs769449 (chr19:45410002)	Aβ deposition	0.39	1.51 × 10^−25^	7.65 × 10^−23^	7.29 × 10^−8^	0.260
	AD-Cm	− 0.06	2.78 × 10^−6^	2.82 × 10^−3^		3.08 × 10^−3^
	PCC-Cm	− 0.10	8.58 × 10^−9^	4.36 × 10^−6^		0.052
	AD-Ct	− 0.11	2.16 × 10^−9^	1.09 × 10^−6^		0.022
	Hv	− 0.73	3.72 × 10^−13^	1.88 × 10^−10^		0.210
rs405509 (chr19:45408836)	Aβ deposition	− 0.09	5.78 × 10^−3^	0.84	1.94 × 10^−3^	0.020
	AD-Cm	0.03	3.15 × 10^−3^	0.40		0.029
	PCC-Cm	0.03	0.017	0.83		0.032
	AD-Ct	0.02	0.16	0.99		0.011
	Hv	0.14	0.089	0.96		0.017

Conditional analysis was performed to test if the influence of these *APOE* variants on neuroimaging features is causal to ADD risk (Table 2). Adjusting for each brain imaging feature effectively reduced the association between the two SNVs (rs429358 and rs769449) and AD susceptibility by approximately 10^5^- to 10^8^-fold in *P* values. This result suggests that the *APOE* variants modulate neuroimaging features that contributes to AD susceptibility. Conversely, the significance of another variant (rs405509) did not change after conditioning, indicating that brain imaging features are independent of its association with AD susceptibility.

### Gene-level analysis for rare variants

**Fig. 4:**
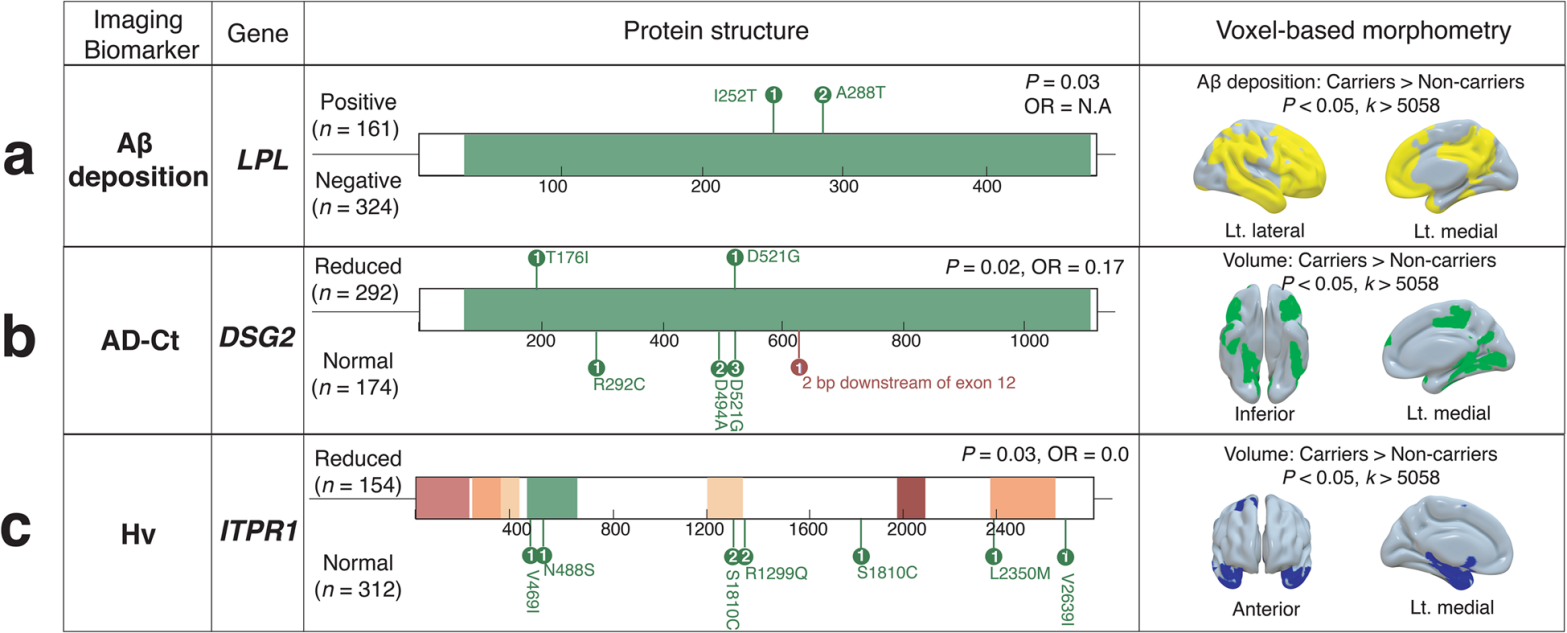
Genes with rare variants that are significantly associated with in vivo AD pathologies. Observed rare functional variants in the case or control groups defined by each clinical parameter are shown for each gene. **a** *LPL* with Aβ deposition in the global brain regions. **b** *DSG2* with AD-signature cortical thickness. **c** *ITPR1* with hippocampal volume. The right panel displays the exploratory voxel-based analyses of brain imaging to demonstrate the regional pattern differences in AD imaging biomarker phenotype between carriers and non-carriers

To investigate the associations of rare variants with AD imaging biomarkers and cognitive impairment, we collected functional rare variants (AF < 0.05, LoF or evolutionary conserved missense variants). A total of eight genes appeared to be significant at a gene-level analysis (*P* < 0.05; Fig. 4; Supplementary Table S6 and Supplementary Fig. S5). Variants in *LPL* were detected only in cases with high Aβ deposition (*n* = 3, *P* = 0.03; Fig. 4a). No rare variants were significantly associated with AD-Cm hypometabolism, but *FERMT2* (OR 2.37 [1.07–5.61]; *P* = 0.02) and *NFAT5* (OR 4.24 [1.15–23.32]; *P* = 0.02) showed significant enrichment for the presence of PCC hypometabolism (Table. S5). *DSG2* showed significant enrichment in controls with normal AD-Ct and Hv (OR 0.17 [0.02–0.89], *P* = 0.02, and OR 0.0 [0.00–1.02], *P* = 0.03; Fig. 4b; Supplementary Fig. S5a). Interestingly, the observed rare variants were mainly located in the sites that bind calcium ions or other proteoglycans, such as *N*-acetylglucosamine (Supplementary Fig. S6c). Also, functional variants in *ITPR1* were observed only in controls with normal Hv (*n* = 9; Fig 4c). These results demonstrate novel associations between rare variants and brain features that can be documented by imaging techniques.

## Discussion

We investigated the associations between genetic variants and various brain features that are accessible via imaging techniques. We identified a group of variants that are linked to Aβ deposition, AD-Cm, PCC-cm, AD-Ct, and Hv (Fig. 1b). Our results bolster the role of *APOE* alleles in contributing to AD pathogenesis susceptibility.

The association analysis of common variants led to the discovery of seven significant associations with AD brain imaging biomarkers. Particularly, we observed an association between *CASS4* common variants and Hv (Table 1). Previous association studies suggested that *CASS4*, encoding a cytoskeletal protein that provides a physical connection with the extracellular matrix, contributes to the risk of ADD [8, 27]. Our observation implies that the previously reported association between *CASS4* and ADD may be through neurodegeneration of the hippocampus via an Aβ-independent pathway. Voxel-wise analysis of MRI demonstrated a region-specific GM atrophy in the bilateral hippocampi and adjacent regions in *CASS4* variant carriers (Fig. 2d). The presence of hippocampus-specific H3K27me3 signals (Supplementary Fig. S3a) suggests that these variants are related to a region-specific epigenetic modification in the hippocampus, although future studies will be necessary to clarify the function of these *CASS4* variants.

*PIWIL1* common variants are related to both Aβ deposition and atrophy in the AD-signature region and the hippocampus (Table 1 and Supplementary Table S7). Voxel-wise analysis of PiB-PET consistently revealed that *PIWIL1* variant carriers displayed greater levels of cerebral Aβ deposition as compared to non-carriers, and regional GM atrophy mainly in the bilateral temporal and PC-PCC areas and medial and inferior frontal regions, which were reported to be vulnerable to the AD process [19]. *PIWIL1* is a member of the PIWI subfamily that plays important roles in stem cell self-renewal, RNA silencing, translational regulation, and neuron development [29], which potentially implicates multiple aspects of brain degeneration. Moreover, the *PIWIL1* variants also co-localize with a brain-specific H3K27me3 signal (Supplementary Figure S3b), which may provide insight into the underlying function of *PIWIL1* in neurodegeneration of AD-vulnerable regions. Additionally, significant associations between common variants in *PSEN1* and *PSEN2* and neurodegeneration biomarkers of AD (i.e., *PSEN1-*rs7523 with AD-Ct and *PSEN2-*rs75733498 with AD-Cm) were observed. Pathogenic rare variants in these genes cause early-onset familial AD. These genes encode major components of the γ-secretase of APP synthesis and proteolysis, leading to Aβ production [30]. It is not clear how these variants are associated with AD-type neurodegeneration, but this observation demonstrates that dysregulation of the genes confers susceptibility to AD via alterations of regional cerebral glucose metabolism or regional cortical thickness, which demonstrates their versatile roles in AD pathogenesis beyond altering Aβ production.

Unlike common or rare variants with association, we found associations between *APOE* variants and all brain imaging features of AD. This finding is consistent with previous reports [31–35], conferring a partial explanation for the genetic link between *APOE* and AD susceptibility. An association test of a variant in the other LD block revealed a marginal association, suggesting a specific functional association between the cell surface receptor binding region and the imaging features. Conditional analysis demonstrated a causal relationship between the imaging features and the association of *APOE* variants with AD susceptibility (Table 2).

The gene-level rare variant association test revealed several genes previously related to AD brain features with cognitive impairment. Notably, carriers of *DSG2* rare variants harbor increased AD-Ct, Hv, and cognitive function, suggesting that these variants play protective roles in neurodegeneration in AD-related regions (Fig 4b and Supplementary Fig. S5a). *DSG2* encodes a calcium-binding glycoprotein components of the desmosome that binds to plaque proteins and intermediate filaments [36] and was reported to confer AD risk based on GWAS [8]. Although a molecular link explaining this association remains unclear, we observed that the variants were located in calcium ion or proteoglycan binding sites (Supplementary Fig. S5c). These sites are evolutionally conserved, and the binding of these molecules is essential for protein function [37].

The KBASE protocol ensures unified subject assessment and standardization of all samples and data collection, processing, and quality control. It enables multi-trait analysis of genetic loci, explicitly targeting each in vivo AD pathology rather than relying on clinical diagnoses with limited accuracy.

### Strengths and limitations

All individuals enrolled in the KBASE cohort underwent careful clinical and genetic characterization. The KBASE protocol adopts unified subject assessment, standardization of all imaging, biofluid, DNA and RNA data collection, and processing followed by meticulous data quality control. We analyzed variants of wide range of allele frequencies on AD-associated genes. Therefore, 132 AD-associated genes for LOAD were covered for genetic variant associations with the AD brain imaging features. Our next step will be to scrutinize the molecular biological mechanisms of variants and their functions on AD pathology.

Several limitations of our study warrant discussions. First, we tried to replicate our results using the ADNI database, which includes unified clinical information, brain imaging data, and genomic data of participants. We adjusted raw brain imaging data of ADNI database using our methodology and conducted association analyses. However, replication was not feasible due to the allele frequency differences originated from the ethnic discrepancies and differences in phenotyping methods, except the APOE SNVs. We also collected a large-scale elderly Korean cohort (*n* = 4683) and conducted a sequencing analysis using our customized targeted LOAD gene panel. Associations between FDG uptake and two novel SNPs were replicated—rs75733498 on *PSEN2* (*P* = 1.6 × 10^−4^) and rs2722372 on *NME8* (*P* = 8.6 × 10^−3^)—although this cohort lacks brain imaging assessment and cognitive impairment trait was accessible.

## Conclusions

Here, we explored the KBASE data and conducted integrative analysis that revealed novel associations between AD-related gene variants and various brain features. Thus, scrutinizing the biological mechanisms of genetic variants and their functions in AD pathology by interrogating multiple aspects of brain morphology and in vivo AD pathologies will lead to a better understanding of the mechanism of AD pathogenesis.

## Supplementary Information

**Additional file 1.** Supplementary methods, tables, and figures.

## Data Availability

All data needed to evaluate the conclusions in the paper are present in the paper. Additional data related to this paper may be requested from the authors.
