# Supplementary material for: Genetic associations of in vivo pathology influence Alzheimer’s disease susceptibility
Source: Alzheimers Res Ther. 2020 Nov 19;12:156. doi: 10.1186/s13195-020-00722-2 (PMC7678113; doi:10.1186/s13195-020-00722-2)
Supplement: Supplementary file 1 — Additional file 1. Supplementary methods, tables, and figures. [file 13195_2020_722_MOESM1_ESM.docx]

**Additional information**

**Genetic associations of *in vivo* pathology influence Alzheimer’s disease susceptibility**

Jieun Seo^1,†^, Min Soo Byun^2,†^, Dahyun Yi^3^, Jun Ho Lee^4^, So Yeon Jeon^5^, Seong A Shin^6^, Yu Kyeong Kim^6^, Koung Mi Kang^7^, Chul-Ho Sohn^7^, Gijung Jung^8^, Jong-Chan Park^1,9^, Sun-Ho Han^1,9^, Jayoung Byun^10^, Inhee Mook-Jung^1,9^, Dong Young Lee^3,7,11,^*, Murim Choi^1,^*; for the KBASE Research Group^‡^

^1^Department of Biomedical Sciences, Seoul National University College of Medicine, Seoul, Republic of Korea

^2^Department of Neuropsychiatry, Seoul National University Bundang Hospital, Gyeonggi, Republic of Korea

^3^Institute of Human Behavioral Medicine, Medical Research Center Seoul National University, Seoul, Republic of Korea

^4^Department of Neuropsychiatry, National Center for Mental Health, Seoul, Republic of Korea

^5^Department of Psychiatry, Chungnam National University Hospital, Daejeon, Republic of Korea

^6^Department of Nuclear Medicine, SMG-SNU Boramae Medical Center, Seoul, Republic of Korea

^7^Department of Radiology, Seoul National University Hospital, Seoul, Republic of Korea

^8^Department of Neuropsychiatry, Seoul National University Hospital, Seoul, Republic of Korea

^9^Department of Biochemistry, Seoul National University College of Medicine, Seoul, Republic of Korea

^10^Department of Medicine, Pusan National University, Busan, Republic of Korea

^11^Department of Psychiatry, Seoul National University College of Medicine, Seoul, Republic of Korea

^†^Equal contributions

*Corresponding authors

^‡^The coinvestigators of the KBASE Research Group are listed elsewhere (http://kbase.kr).

**Supplementary Methods**

**Table S1.** Characteristics of the KBASE cohort used this study

**Table S2.** Definition of AD imaging biomarkers used in this study

**Table S3.** Gene list in the AD panel

**Table S4.** Data quality of 557 sequencing runs

**Table S5.** Parameters used in the association tests

**Table S6.** List of genes with rare variants that were associated with brain imaging biomarkers

**Table S7.** Association of significant SNVs with all brain imaging biomarkers

**Figure S1.** Evaluating our Ion Torrent sequencing data variant calling method

**Figure S2.** Additional common variants associated with brain imaging features

**Figure S3.** H3K27me3 signals display epigenetic implications in brain imaging feature-associated loci

**Figure S4.** Prediction of AD susceptibility by the function of association of common variants with *in vivo* AD imaging features

**Figure S5.** Additional genes with rare variants that display associations with the imaging features

This supplementary material has been provided by the authors to give readers additional information about their work.

**Supplementary Methods**

***Participants of the KBASE cohort***

This study was performed in part of the Korean Brain Aging Study for the Early Diagnosis and Prediction of Alzheimer’s Disease (KBASE), an ongoing prospective cohort study started in 2014 in Seoul, Korea. Detailed information on the recruitment, methodologies and baseline sample characteristics of the KBASE cohort is provided in the previous study.[1] This study recruits individuals of cognitively normal (CN), mild cognitive impairment (MCI), and AD dementia, according to the following criteria.

1. CN young and middle-aged adults (CN-ym) group: (a) age 20-54 years (inclusive), (b) Clinical Dementia Rating (CDR) score of 0, and (c) no diagnosis of MCI or dementia.
2. CN older adults (CN-old) group: (a) age 55-90 years (inclusive), (b) CDR score of 0, and (c) no diagnosis of MCI or dementia.
3. MCI group: (a) age 55-90 years (inclusive), (b) individuals who met the core clinical criteria for diagnosis of MCI according to the recommendations of the National Institute on Aging and Alzheimer’s Association (NIA-AA) guidelines, *i.e.*, (i) memory complaint corroborated by self, an informant, or clinician, (ii) objective memory impairment for age, education, and gender, (iii) largely intact functional activities, and (iv) not demented, and (c) a global CDR score of 0.5.
4. AD dementia group: (a) age 55-90 years (inclusive), (b) individuals who met the criteria for dementia in accordance with the Diagnostic and Statistical Manual of Mental Disorders 4^th^ Edition (DSM-IV-TR) and the criteria for probable AD dementia in accordance with the NIA-AA guidelines, and (c) a global CDR score of 0.5 or 1.

Exclusion criteria for all participants are: (1) presence of major psychiatric illness (*e.g.*, schizophrenia, bipolar disorder, major depressive disorder, alcohol/substance abuse or dependence and delirium), (2) significant neurological or medical condition or comorbidities that could affect mental functioning, (3) contraindications for MRI scan (*e.g.*, pacemaker, claustrophobia), (4) illiteracy, (5) presence of significant visual/hearing difficulty, severe communication or behavioral problems that would make a clinical examination or brain scan difficult, (6) taking an investigational drug, and (7) in pregnancy or breastfeeding.

***Image acquisition and preprocessing for measurement of in vivo AD pathologies***

**[^11^C]Pittsburg compound B (PiB) – positron emission tomography (PET) image acquisition and preprocessing**

Participants underwent simultaneous three-dimensional (3D) PiB-PET and 3D T1-weighted magnetic resonance (MR) imaging using a 3.0T Biograph mMR scanner (PET-MR scanner; Siemens, Washington DC) according to the manufacturer’s approved guidelines. After intravenous administration of 555 MBq of [^11^C]PiB (range, 450-610 MBq), a 30-min emission scan was obtained 40 min after injection. The PiB-PET data collected in list mode were processed for routine corrections such as uniformity, UTE-based attenuation, and decay corrections, and were reconstructed into a 256 x 256 image matrix using iterative methods (6 iterations with 21 subsets).

The following image preprocessing steps were performed using Statistical Parametric Mapping 8 (SPM8; http://www.fil.ion.ucl.ac.uk/spm) implemented in Matlab 2014a (Mathworks, Natick, MA). Static PiB-PET images were co-registered to individual T1 structural images and transformation parameters for the spatial normalization of individual T1 images to a standard Montreal Neurological Institute (MNI) template were calculated. Using IBASPM software, we used the inverse transformation parameters to transform coordinates from the automatic anatomic labeling (AAL) 116 atlas[2] into an individual space for each subject (a resampling voxel size = 1 x 0.98 x 0.98 mm). In addition, a gray matter mask was applied for each individual, to extract gray matter and exclude the non-gray matter portions of the atlas (*i.e.* white matter and cerebrospinal fluid space).

The mean regional [^11^C]PiB uptake values from cerebral regions were extracted using the individual AAL116 atlas from T1-coregistered PiB-PET images. Cerebellar gray matter was used as the reference region for quantitative normalization of cerebral PiB uptake values due to its relatively low Aβ deposition.[3] To measure PiB uptake in the cerebellar gray matter regions, a probabilistic cerebellar atlas (Institute of Cognitive Neuroscience, UCL; Cognitive Neuroscience Laboratory, Royal Holloway) was transformed into individual space in the same manner as described above. Of the 28 anatomical structural regions in the cerebellar atlas, all cerebellar lobular regions except the vermis were included to extract the mean cerebellar uptake values.

We used regional definition of Aβ positivity based on previous studies that characterized participants as Aβ positive if measurements of PiB uptake exceeded a minimum threshold in at least one regions-of-interest (ROI).[4, 5] The automatic anatomic labeling algorithm and a region combining method[5] were applied to determine ROIs to characterize the [^11^C]PiB retention level in the frontal, lateral parietal, posterior cingulate-precuneus, and lateral temporal regions. The standardized uptake value ratio (SUVR) values for each ROI were calculated by dividing the mean value for all voxels within each ROI by the mean cerebellar uptake value in the same image.

Each participant was classified as Aβ positive if the SUVR value was > 1.4 in at least one of the four ROIs or as Aβ negative if the SUVR values of all four ROIs was ≤ 1.4, and a global Aβ retention value was generated by dividing the mean value for all voxels of the global cortical ROI consisting of the four ROIs by the mean cerebellar uptake value in the same image.[5, 6]

**[^18^F]Fluorodeoxyglucose (FDG)-PET image acquisition and preprocessing**

The participants fasted for at least 6 hours and rested in a waiting room for 40 min prior to the scans after intravenous administration of 0.1 mCi/Kg of [^18^F]FDG radioligands. The PET data collected in list mode (5 min x 4 frames) were processed for routine corrections such as uniformity, UTE-based attenuation, and decay corrections. After inspecting the data for any significant head movements, we reconstructed them into a 20-min summed image using iterative methods (6 iterations with 21 subsets).

The following image processing steps were performed using SPM12 (http://www.fil.ion.ucl.ac.uk/spm) implemented in Matlab 2014a. First, static FDG-PET images were co-registered to individual T1 structural images, and transformation parameters for the spatial normalization of individual T1 images to a standard MNI template were calculated and used to spatially normalize the PET images to the MNI template. After smoothing the spatially normalized FDG-PET images with a 12-mm Gaussian filter, intensity normalization was performed using the pons as the reference region.

Voxel-weighted mean SUVRs of FDG uptake in the AD-signature FDG ROIs consisting of the bilateral angular gyri, PC-PCC, and inferior temporal gyri.[7] AD-signature hypometabolism positivity was also defined if a mean SUVR of AD-signature FDG ROIs < 1.475. This cut-off was determined based on Youden index calculated from receiver operating characteristic (ROC) curve analyses to determine the optimal threshold that can distinguish the ADD group (*n* = 58) from the CN group (*n* = 260) in the KBASE cohort (Sensitivity = 91.4%). In addition, as PCC is the region affected from the very early stage of AD,[8] we also calculated a mean SUVR of FDG uptake in the PCC and a binary variable for the positivity of hypometabolism in the PCC obtained via abovementioned ROC curve analyses (Cut-off for PCC = 1.543, Sensitivity = 91.4%).

**MR image acquisition and preprocessing**

All T1-weighted images were acquired in the sagittal orientation using the abovementioned 3.0T PET-MR machine. MR image acquisition parameters were as follows: repetition time = 1,670 ms, echo time = 1.89 ms, field of view 250 mm, and 256 x 256 matrix with 1.0-mm slice thickness. All MR images were automatically segmented using FreeSurfer version 5.3 (http://surfer.nmr.mgh.harvard.edu/) with manual correction of minor segmentation errors.

Based on the Desikan–Killiany atlas,[9] cortical thickness in the AD-signature regions (AD-Ct) was defined as mean cortical thickness values were obtained from AD-signature regions, including the entorhinal, parahippocampal, middle temporal, angural gyri, precunues and PCC according to a previous study.[10] Presence of cortical atrophy in the AD-signature region was defined if AD-Ct was < 2.457 mm, the cut-off which distinguished ADD groups (*n* = 52) from CN groups (*n* = 254) with 90% sensitivity.[6]

To obtain intracranial volume (ICV)-adjusted hippocampal volume (Hv), left and right hippocampi were first extracted and added together to yield the total Hv. Then, the volume deviating from the expected total HV according to ICV in the reference group (*i.e.*, young CN group of the study cohort) was calculated to obtain HV.[11] Detailed information on the characteristics of the reference group for Hv were reported previously.[11] The positivity for the hippocampal atrophy was defined if Hv was less than -1,943.617 mm^3^, the cut-off based on threshold distinguishing ADD from the CN groups with 90% sensitivity.[6]

***DNA Library Preparation and Targeted Sequencing***

Genomic DNA was extracted from peripheral blood, quantified using the Qubit Fluorometer and sample purity was checked using Nanodrop (Thermo Fisher Scientific, Waltham, MA). Libraries were generated using Ion AmpliSeq Customized Panel (Thermo Fisher Scientific, Waltham, MA) according to the manufacturer’s recommendations. 10 ng of genomic DNA from each sample was used to prepare barcoded libraries using IonXpress barcoded adapters (Thermo Fisher Scientific). Libraries were combined to a final concentration of 3 ng/ml using the Ion Library Quantification Kit (Thermo Fisher Scientific), and emulsion PCR was performed using the Ion Torrent OneTouchTM 2 System. Samples were sequenced on the Ion Proton sequencer (Thermo Fisher Scientific) using Ion P1 chips (eFigure 1 and eTable 4 in the Supplement).

***Pilot experiment to optimize variant calling of Ion torrent sequencing data.***

We conducted pilot experiments to determine the variant calling criteria. We used our Ion Torrent AD target panel to sequence six samples, which were also whole-exome sequenced on the Illumina platform. Using the AD panel region variants from the WES as gold-standard, the heterozygous variant MAF interval that yielded the highest sensitivity and specificity was determined. As a result, 92.4% sensitivity and 93.5% specificity were obtained when 0.35-0.65 cutoff for heterozygous variants and 0.95-1.0 for homozygous variants were used (eFigure 1A). To further test if our genotype calling is consistent with other studies, common variant AF in KBASE with those from individuals of the 1000 Genome Project were compared (eFigure 1B). Overall correlation with all individuals was strong (*r* = 0.94), especially with East Asian population (*r* = 0.99).

***Regional plots***

LocusZoom (http://locuszoom.sph.umich.edu), a web-based software, was used to plot the common SNV-level association test result.

***H3K27me3 ChIP-seq data analysis***

To elucidate epigenetic implication of significantly associated loci with *in vivo* AD pathology, we downloaded call sets from the ENCODE portal (https://www.encodeproject.org/) with the following identifiers: ENCFF812HOX, ENCFF514NSO, ENCFF514NSO, ENCFF113WPZ, ENCFF366HAW, ENCFF042AXG and ENCFF369IKU.

***AD susceptibility prediction score with imaging biomarkers***

We quantified the genetic liability of common variants significantly associated with imaging biomarkers (*P* < 0.05) in predicting of AD susceptibility. Risk scores for each individual with each imaging feature were calculated as a weighted sum of the common variants using effect sizes (odds ratios) from association analysis. The total score ranges were divided into ten intervals, and portion of individuals with cognitive impairment in each decile was calculated. To evaluate an integrated effect of multi-imaging features, all the variants associated with at least one imaging features were used.

***Exploratory voxel-based analysis of multi-modal brain imaging***

For a demonstration purpose, exploratory voxel-based analyses of each image modality (PiB-PET, FDG-PET and MRI) were performed between variants carriers and noncarriers using Statistical Parametric Mapping 12 (SPM12; http://www.fil.ion.ucl.ac.uk/spm) implemented on Matlab R2017b. For common variants analysis, ANCOVA was performed to compare between variants carriers and noncarriers after adjusting the effect of age, sex and number of *APOE4* allele. Results were initially examined at *P* < 0.01 according to the previous study[12] and multiple comparisons were corrected based on cluster-correction procedure using Analysis of Functional NeuroImage (*i.e.*, 3dClustSim, version built on Feb 10^th^, 2017) with 10,000 iterations of Monte Carlo simulations on an anatomical cerebral mask dataset with 1,801,748 voxels (as analysis for this study was restricted to the cerebrum. Cerebellum was excluded).[13] Thus, significant clusters after multiple comparison correction were reported at uncorrected *P* < 0.01, and *k* > 1,497 voxels. For *GORASP1*, a voxel-based analysis result of PiB-PET was reported at uncorrected *P* < 0.05, and *k* > 5,058 for a demonstrative purpose.

For gene-level tests for rare variants, randomly selected controls were compared with cases carrying rare variants in 1:5 ratio to correct for small sample size of cases carrying rare variants using independent t-test. Exploratory voxel-wise analysis to compare cases and controls of each gene with rare variants were performed and results were reported at uncorrected *P* < 0.05 and *k* > 5,058.

**Table S1.** Characteristics of the KBASE cohort used this study. Data are presented as mean (SD) or percentages.

|  | Total | ADD | MCI | CN-old | CM-ym |
| --- | --- | --- | --- | --- | --- |
| **Sample size** | 557 | 84 | 137 | 274 | 62 |
| **Age (years)** | 67.4 (13.4) | 72.8 (8.2) | 73.7 (7.0) | 69.4 (8.0) | 37.6 (9.7) |
| **Sex (females)** | 322 (57.8%) | 58 (69.0%) | 90 (65.7%) | 139 (50.7%) | 35 (56.5%) |
| ***APOE4* allele freq. (%)** | 16.6% | 35.7% | 20.4% | 9.3% | 14.3% |
| ***APOE4* carriers** | 160 (28.7%) | 49 (58.3%) | 46 (33.6%) | 49 (17.9%) | 16 (25.8%) |
| **CDR (0/0.5/1)** | 336/165/56 | 0/28/56 | 0/137/0 | 274/0/0 | 62/0/0 |
| **Family history^a^** | 109 (19.6%) | 17 (20.2%) | 32 (23.4%) | 56 (20.4%) | 4 (6.5%) |
| **Education (years)** | 11.2 (4.9) | 9 (5.2) | 10.0 (4.5) | 11.7 (4.8) | 15.0 (1.9) . |

*^a^* Family history of AD dementia in the 1^st^ degree relatives

**Table S2.** Definition of AD imaging biomarkers used in this study

| AD Imaging biomarker | Type of variable | Description | Common variant association test | Rare variant  gene-based test |
| --- | --- | --- | --- | --- |
| 1. Cerebral Amyloid-beta accumulation measured by PiB-PET | | | | |
| Aβ deposition | Quant. | Voxel-weighted mean SUVR of PiB uptake in the cortical global ROIs consisting of frontal, lateral parietal, precunues-posterior cingulate (PCC) and lateral temporal regions | Y | - |
|  | Bin. | If the SUVR value was > 1.4 in at least one of the four ROIs | Y | Y |
| 1. Glucose metabolism levels measured by FDG-PET | | | | |
| AD-Cm | Quant. | Voxel-weighted mean SUVR of FDG uptake in the AD-signature FDG-ROIs sensitive to AD process[7] | Y | - |
|  | Bin | AD-Cm hypometabolism positive If mean SUVR of AD-signature FDG-ROI < 1.475 | Y | Y |
| PCC-Cm | Quant. | Mean SUVR of FDG uptake in the PCC | Y | - |
|  | Bin | PCC hypometabolism positive If mean SUVR of PCC < 1.543 | Y | Y |
| 1. Cortical thickness measured by MRI | | | | |
| AD-Ct | Quant. | Mean cortical thickness in the AD-signature regions according to the previous study[10] | Y | - |
|  | Bin | Cortical atrophy of AD-signature region If AD-Ct < 2.457 mm | Y | Y |
| 1. Hippocampal volume reduction measured by MRI | | | | |
| Hv | Quant. | Intracranial volume-adjusted hippocampal volume | Y | - |
|  | Bin | Hippocampal atrophy positive If Hv < -1,943.617 mm^3^ | Y | Y |

**Table S3.** Gene list in the AD panel

| **Source** | **Gene symbol** | **Number of genes** |
| --- | --- | --- |
| GWAS | *ABCA7, POLR2E, CNN2, BIN1, CASS4, CD2AP, CD33, NR1H3, DDB2, MADD, PTPMT1, NDUFS3, CLU, CR1, DSG2, EPHA1, ZYX, FERMT2, INPP5D, MEF2C, MS4A4A, MS4A6A, MS4A6E, NME8, GPR141, PICALM, PTK2B, RIN3, SLC24A4, SORL1, NYAP1, ARID5B* | 32 |
| OMIM | *CALHM1, ACE, ADAM10, APOE, APP, ATXN1, MAPT, PSEN1, PSEN2, TREM2, A2M, APBB1, APBB2, CD36, HDAC6, PLAU, PPARG, SIRT1, UBQLN1* | 19 |
| KEGG | *APH1A, PSENEN, APOBEC3B, ARNTL, BACE1, BACE2, CASP3, CASP7, CASP8, CASP9, CDK5, CDK5R1, CREBBP, FADD, FAS, GAPDH, GNAQ, GRIN1, GRIN2A, GRIN2B, GRIN2C, GRIN2D, GSK3B, IDE, IL1B, ITPR1, ITPR2, ITPR3, LPL, LRP1, MAPK1, MAPK3, MME, NCSTN, NOS1, PTGS1, RYR3, SNCA, TLR2, TNF, TLR4, TNFRSF1A, NFAT5, CLOCK, PER2, PIWIL1, PIWIL2, PIWIL3, PIWIL4, TARDBP, TERT, PLD3, SIRT6* | 53 |
| Custom selection | *REST, SQSTM1, TFEB, ERN1, BECN1, GORASP2, GORASP1, LRRK1, LRRK2, SEC23A, SEC23B, SAR1A, MECP2, SOX2, CREB1, HDAC1, RAI1, PIK3CA, RPS6KA3, AKT3, ASTN2, AGER, EPHB2, PRNP, NR1D1, LILRB2, FCGR2B, GAD2* | 28 |

**Table S4.** Data quality of 557 sequencing runs

|  | Mean | s.d. |
| --- | --- | --- |
| Mean read lengths (bp) | 166.76 | 9.96 |
| # of reads per run (millions) | 2.64 | 0.75 |
| Mean coverage depth (X) | 436.82 | 118.66 |
| % of reads on genome | 99.54 | 0.21 |
| % of reads on target | 91.50 | 0.63 |
| % of targeted bases covered at least 4x | 99.11 | 0.36 |
| % of targeted bases covered at least 8x | 98.71 | 0.43 |
| % of targeted bases covered at least 20x | 97.77 | 0.70 |
| % of targeted bases covered at least 50x | 95.55 | 1.59 |
| % of targeted bases covered at least 100x | 90.33 | 4.04 |

**Table S5.** Parameters used in the association tests

| Test | Trait type | Regression model | Covariates | Multiple test correction method used |
| --- | --- | --- | --- | --- |
| Common SNV | Quantitative | Linear, Additive | Gender,  age and  *APOE* E4 allele number | None |
|  | Binomial | Logistic, Additive |  |  |
| Rare SNV | Quantitative | Linear, Additive |  |  |
|  | Binomial | Logistic, Additive |  |  |
| APOE | Quantitative | Linear, Additive | Gender and age | The Benjamini–Hochberg method |
|  | Binomial | Logistic, Additive |  |  |

**Table S6.** List of genes with rare variants that were associated with brain imaging biomarkers (*P* < 5.0 x 10^-2^, Fisher’s exact test).

| AD imaging biomarker | Gene | No. of cases | | No. of controls | | Ratio of  LoF  (Case/control) | Ratio of  Ref. allele carriers (Case/control) | *P* | OR |
| --- | --- | --- | --- | --- | --- | --- | --- | --- | --- |
|  |  | Alt.  allele | Ref.  allele | Alt. allele | Ref. allele |  |  |  |  |
| Aβ deposition | *LPL* | 3 | 313 | 0 | 648 | 0 | 0.51 | 0.03 | N.A. |
| PCC-Cm | *FERMT2* | 24 | 466 | 10 | 460 | 2.4 | 1.01 | 0.02 | 2.37 |
|  | *NFAT5* | 13 | 477 | 3 | 467 | 4.33 | 1.02 | 0.02 | 4.24 |
| AD-Ct | *DSG2* | 2 | 582 | 7 | 341 | 0.29 | 1.71 | 0.02 | 0.17 |
| Hv | *DSG2* | 0 | 308 | 9 | 615 | 0 | 0.5 | 0.03 | 0.0 |
|  | *ITPR1* | 0 | 308 | 9 | 615 | 0 | 0.5 | 0.03 | 0.0 |
| Cognitive impairment | *ABCA7* | 12 | 430 | 5 | 543 | 2.40 | 0.79 | 0.05 | 3.03 |
|  | *DSG2* | 1 | 441 | 8 | 540 | 0.13 | 0.82 | 0.05 | 0.15 |
|  | *ERN1* | 14 | 428 | 7 | 541 | 2 | 0.79 | 0.05 | 2.53 |
|  | *FERMT2* | 23 | 419 | 13 | 535 | 1.77 | 0.78 | 0.03 | 2.26 |
|  | *TLR4* | 4 | 438 | 0 | 548 | N.A. | 0.8 | 0.04 | N.A. |

**Table S7.** Association of significant SNVs with all brain imaging biomarkers. *P*-values in grey are original significant associations.

|  |  | Aβ deposition | | AD-Cm | | PCC-Cm | | AD-Ct | | Hv | |
| --- | --- | --- | --- | --- | --- | --- | --- | --- | --- | --- | --- |
| dbSNP ID | Gene | Bin. | Quant. | Bin. | Quant. | Bin. | Quant. | Bin. | Quant. | Bin. | Quant. |
| rs3732377 | *GORASP1* | 9.32 x 10^-4^ | 0.04 | 0.58 | 0.22 | 0.86 | 0.31 | 0.20 | 0.21 | 0.63 | 0.54 |
| rs1109643 | *GORASP1* | 9.79 x 10^-4^ | 0.11 | 0.36 | 0.86 | 0.53 | 0.96 | 0.74 | 0.67 | 0.88 | 0.15 |
| rs28362644 | *GORASP1* | 7.02 x 10^-4^ | 0.10 | 0.35 | 0.80 | 0.42 | 0.85 | 0.74 | 0.68 | 0.79 | 0.16 |
| rs2290149 | *MADD* | 2.02 x 10^-4^ | 0.03 | 0.52 | 0.07 | 0.06 | 0.02 | 0.36 | 0.99 | 0.05 | 0.26 |
| rs75733498 | *PSEN2* | 0.02 | 0.09 | 1.75 x 10^-4^ | 2.03 x 10^-3^ | 2.67 x 10^-3^ | 8.06 x 10^-3^ | 0.59 | 0.19 | 0.54 | 0.08 |
| rs2722372 | *NME8* | 0.74 | 0.91 | 0.03 | 7.63 x 10^-4^ | 0.02 | 5.71 x 10^-4^ | 0.65 | 0.19 | 0.03 | 0.06 |
| rs7523 | *PSEN1* | 0.32 | 0.41 | 0.16 | 0.88 | 0.18 | 0.58 | 1.74 x 10^-5^ | 7.74 x 10^-3^ | 0.34 | 0.45 |
| rs3746623 | *CASS4* | 0.94 | 0.75 | 0.95 | 0.27 | 1.00 | 0.45 | 0.96 | 4.09 x 10^-3^ | 0.75 | 1.73 x 10^-4^ |
| rs3746625 | *CASS4* | 0.94 | 0.63 | 0.95 | 0.31 | 1.00 | 0.46 | 0.91 | 2.84 x 10^-3^ | 0.75 | 1.73 x 10^-4^ |
| rs3746626 | *CASS4* | 0.46 | 0.63 | 0.47 | 0.31 | 0.97 | 0.46 | 0.29 | 2.84 x 10^-3^ | 5.29 x 10^-3^ | 1.73 x 10^-4^ |
| rs4811697 | *CASS4* | 0.46 | 0.63 | 0.47 | 0.31 | 0.97 | 0.46 | 0.29 | 2.84 x 10^-3^ | 5.29 x 10^-3^ | 3.42 x 10^-4^ |
| rs10848087 | *PIWIL1* | 0.12 | 5.05 x 10^-4^ | 5.05 x 10^-3^ | 0.08 | 0.05 | 0.08 | 0.09 | 2.94 x 10^-4^ | 4.24 x 10^-4^ | 2.31 x 10^-4^ |

**Figure S1. Evaluating our Ion Torrent sequencing data variant calling method.** (a) Distribution of true positive and false positive variants after comparing our calls to Illumina WES calls. (b) Correlations of common variant AF with the 1000 Genome Project (*r*, correlation coefficient).

**
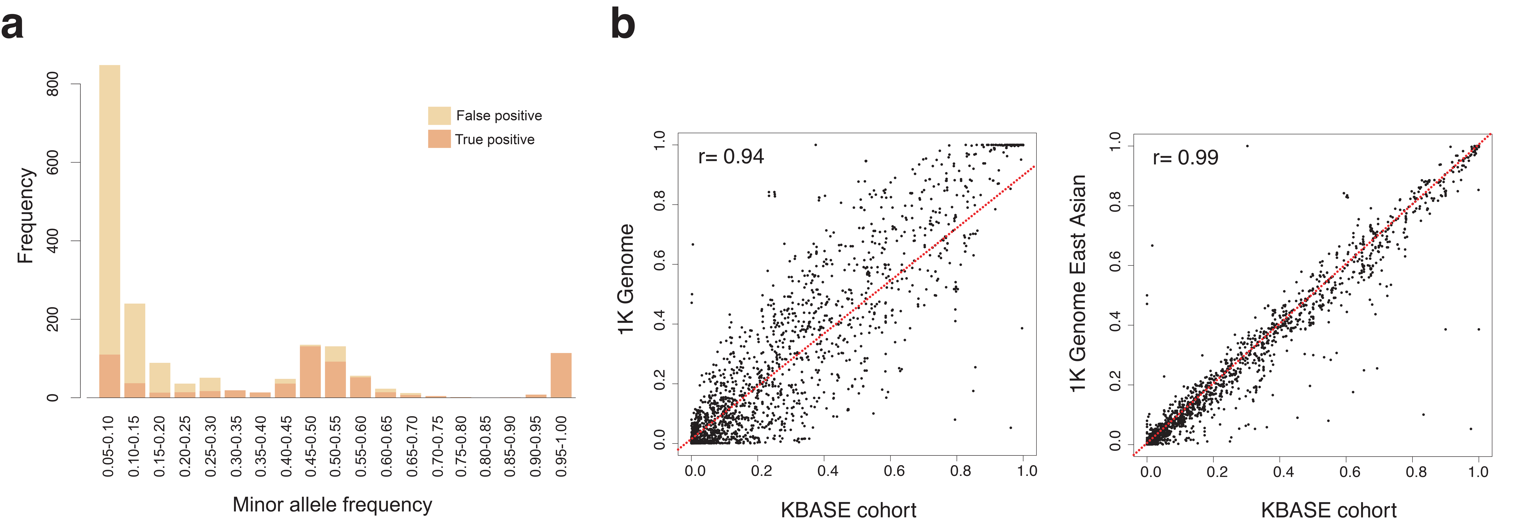
**

**Figure S2. Additional common variants associated with brain imaging features.** (a) rs2290149 on *MADD* and rs28362644 on *GORASP1* with cerebral Aβ deposition. (b) rs73733498 on *PSEN2* with AD-Cm. (c) rs10848087 on *PIWIL1* with AD-Ct (upper) and Hv (lower).


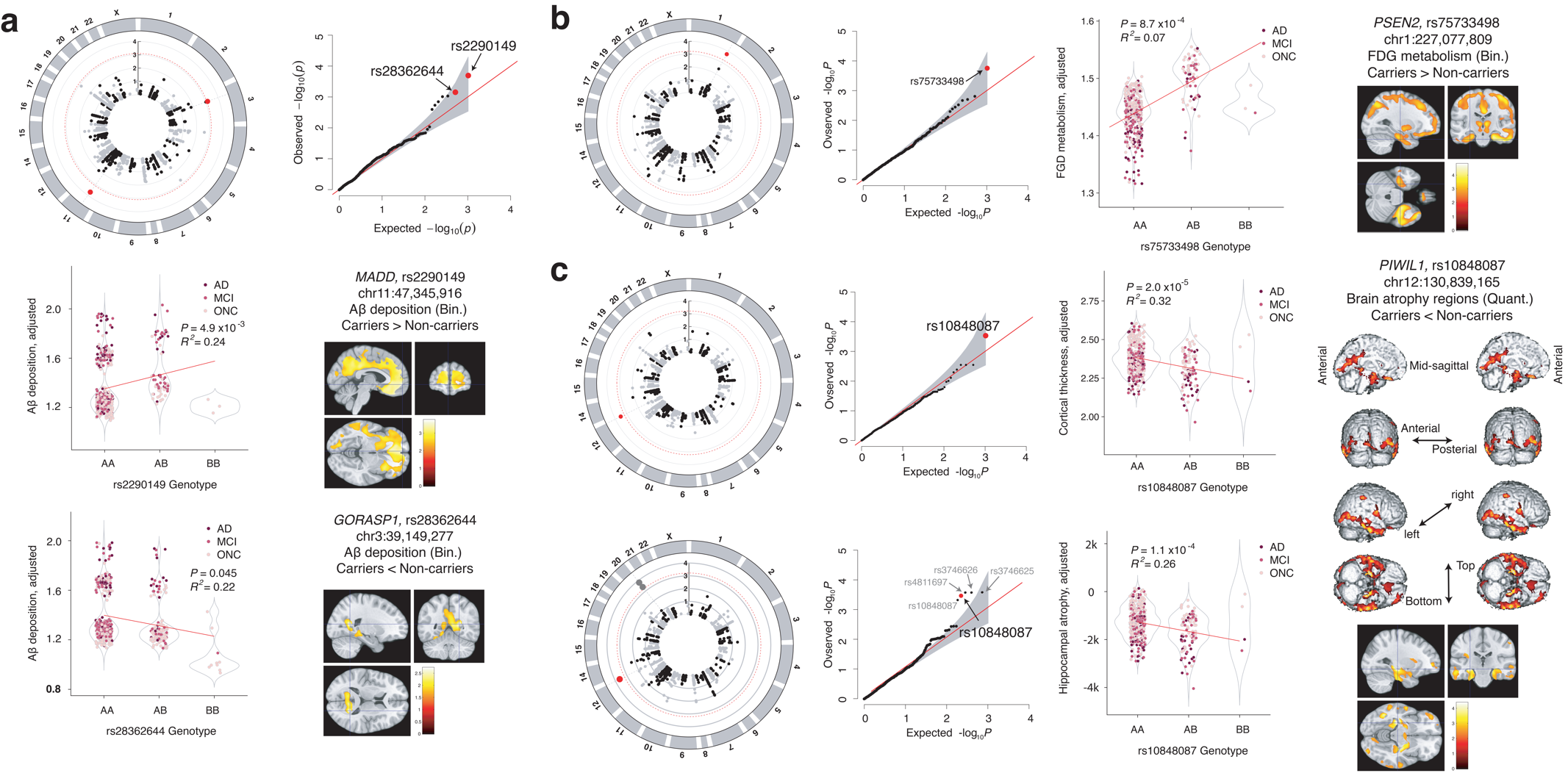


**Figure S3. H3K27me3 signals display epigenetic implications in brain imaging feature-associated loci.** (a) Hippocampus-specific H3K27me3 signal at four significant SNVs on *CASS4*. (b) H3K27me3 signal at rs10848087 on *PIWIL1* in affected brain regions.


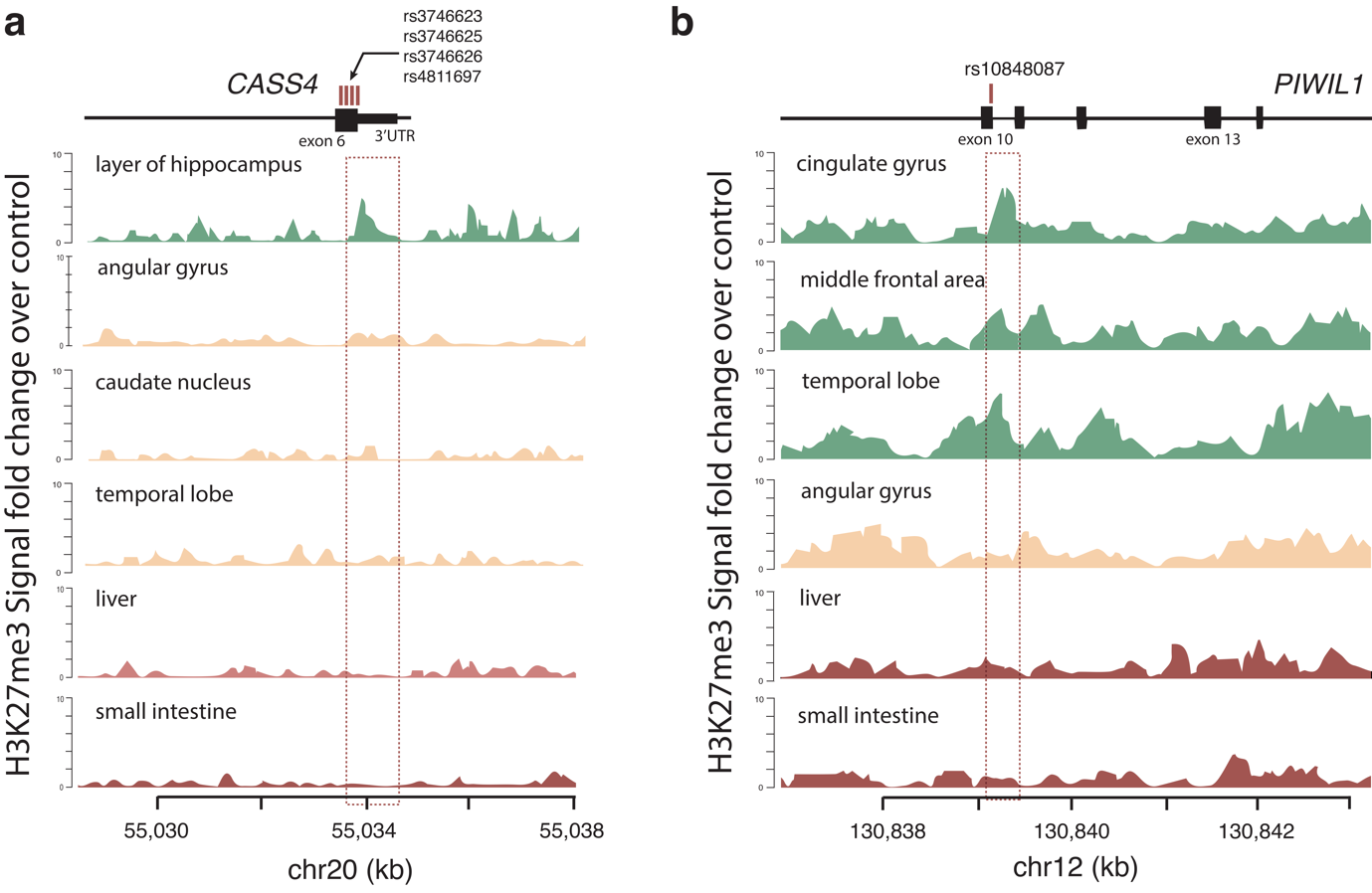


**Figure S4.** **Prediction of cognitive impairment susceptibility by the risk score based on common genetic variants associated with AD imaging features.** Risk score was calculated as a weighted sum of the effect sizes (odds ratios) of common variants associated with each individual imaging feature (a-e) or those associated with any one of all five features (f).


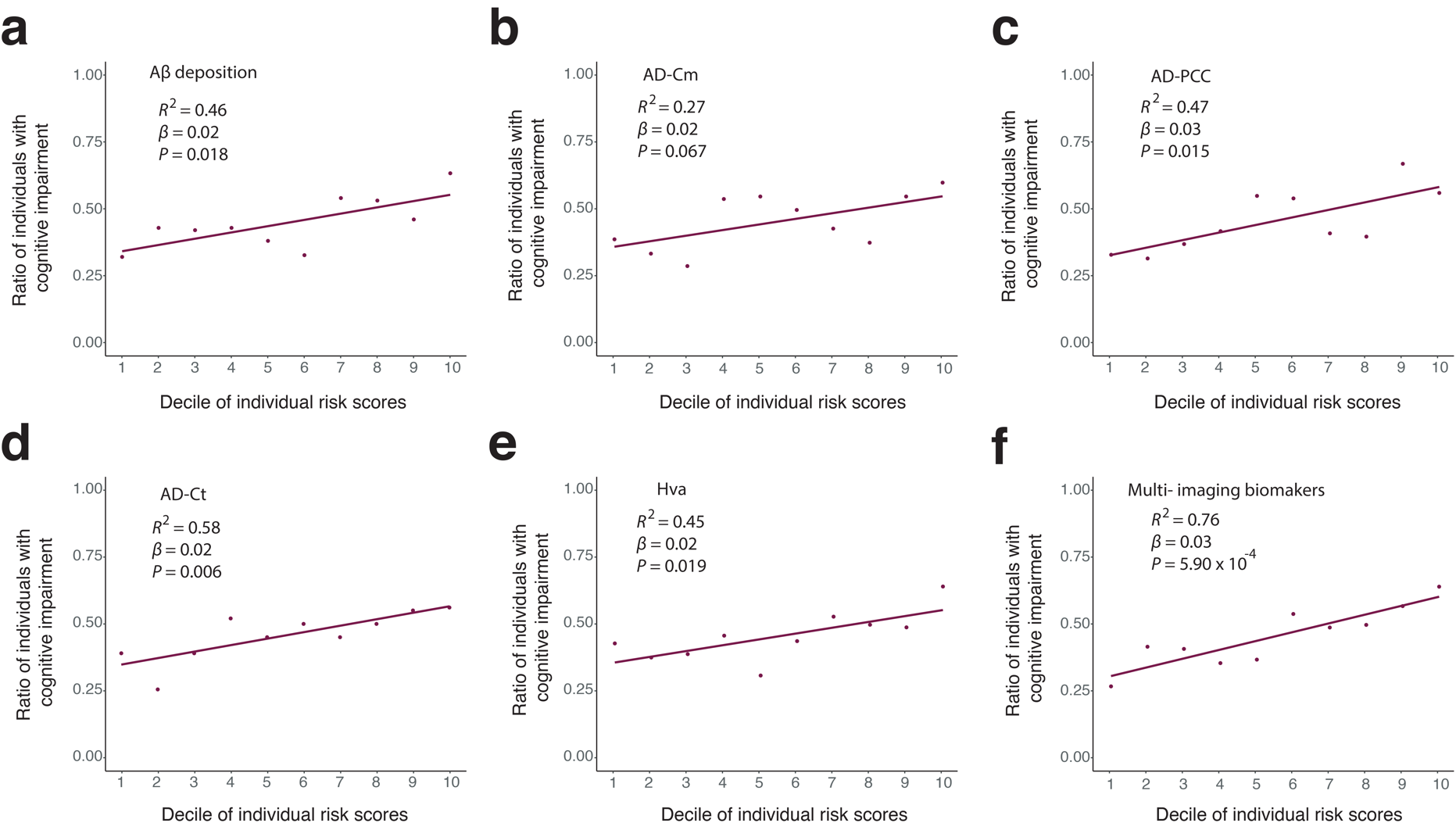


**Figure S5. Additional genes with rare variants that display associations with the imaging features.** (a) *DSG2* with hippocampal volume reduction. (b) *ABCA7, DSG2, ERN1, FERMT2* and *TLR4* with cognitive impairment. (c) Three-dimensional model of the DSG2 homodimer complex with enhanced views of the domains where observed rare variants are located (protein database ID 5ERD). Side chains of the variant amino acids are marked in pink. The pink poly-spheres show mannonanose-di-(N-acetylglucosamine) (MAN), and the beige poly-spheres N-acetylglucosamine (NAG). Green spheres represent Ca^2+^ cations.


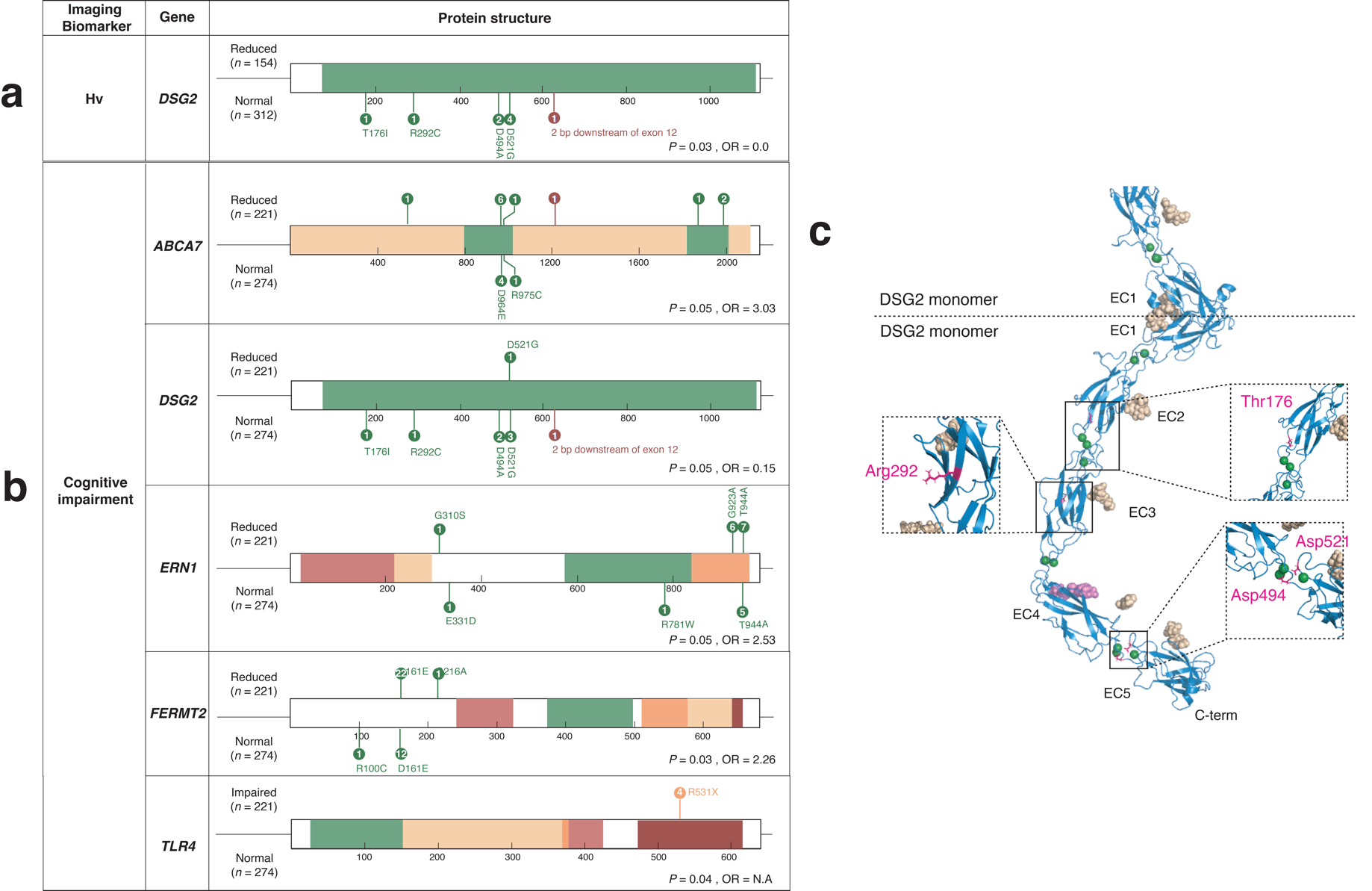


**References**

1. Byun MS, Yi D, Lee JH, Choe YM, Sohn BK, Lee JY, et al. Korean Brain Aging Study for the Early Diagnosis and Prediction of Alzheimer's Disease: Methodology and Baseline Sample Characteristics. Psychiatry Investig. 2017;14(6):851-63.

2. Tzourio-Mazoyer N, Landeau B, Papathanassiou D, Crivello F, Etard O, Delcroix N, et al. Automated anatomical labeling of activations in SPM using a macroscopic anatomical parcellation of the MNI MRI single-subject brain. Neuroimage. 2002;15(1):273-89.

3. Lopresti BJ, Klunk WE, Mathis CA, Hoge JA, Ziolko SK, Lu X, et al. Simplified quantification of Pittsburgh Compound B amyloid imaging PET studies: a comparative analysis. J Nucl Med. 2005;46(12):1959-72.

4. Aizenstein HJ, Nebes RD, Saxton JA, Price JC, Mathis CA, Tsopelas ND, et al. Frequent amyloid deposition without significant cognitive impairment among the elderly. Arch Neurol. 2008;65(11):1509-17.

5. Reiman EM, Chen K, Liu X, Bandy D, Yu M, Lee W, et al. Fibrillar amyloid-beta burden in cognitively normal people at 3 levels of genetic risk for Alzheimer's disease. Proc Natl Acad Sci U S A. 2009;106(16):6820-5.

6. Jack CR, Jr., Wiste HJ, Weigand SD, Rocca WA, Knopman DS, Mielke MM, et al. Age-specific population frequencies of cerebral beta-amyloidosis and neurodegeneration among people with normal cognitive function aged 50-89 years: a cross-sectional study. Lancet Neurol. 2014;13(10):997-1005.

7. Jagust WJ, Landau SM, Shaw LM, Trojanowski JQ, Koeppe RA, Reiman EM, et al. Relationships between biomarkers in aging and dementia. Neurology. 2009;73(15):1193-9.

8. Minoshima S, Giordani B, Berent S, Frey KA, Foster NL, Kuhl DE. Metabolic reduction in the posterior cingulate cortex in very early Alzheimer's disease. Ann Neurol. 1997;42(1):85-94.

9. Desikan RS, Segonne F, Fischl B, Quinn BT, Dickerson BC, Blacker D, et al. An automated labeling system for subdividing the human cerebral cortex on MRI scans into gyral based regions of interest. Neuroimage. 2006;31(3):968-80.

10. Wirth M, Villeneuve S, Haase CM, Madison CM, Oh H, Landau SM, et al. Associations between Alzheimer disease biomarkers, neurodegeneration, and cognition in cognitively normal older people. JAMA Neurol. 2013;70(12):1512-9.

11. Lee JH, Byun MS, Yi D, Choe YM, Choi HJ, Baek H, et al. Sex-specific association of sex hormones and gonadotropins, with brain amyloid and hippocampal neurodegeneration. Neurobiol Aging. 2017;58:34-40.

12. Apostolova LG, Risacher SL, Duran T, Stage EC, Goukasian N, West JD, et al. Associations of the Top 20 Alzheimer Disease Risk Variants With Brain Amyloidosis. JAMA Neurol. 2018;75(3):328-41.

13. Forman SD, Cohen JD, Fitzgerald M, Eddy WF, Mintun MA, Noll DC. Improved assessment of significant activation in functional magnetic resonance imaging (fMRI): use of a cluster-size threshold. Magn Reson Med. 1995;33(5):636-47.
